# ADDP and PS-PPh_3_: an efficient Mitsunobu protocol for the preparation of pyridine ether PPAR agonists

**DOI:** 10.1186/1860-5397-2-21

**Published:** 2006-10-31

**Authors:** Paul S Humphries, Quyen-Quyen T Do, David M Wilhite

**Affiliations:** 1 Pfizer Global R&D, Department of Medicinal Chemistry, 10614 Science Center Drive, San Diego, CA 92121, USA

## Abstract

A series of pyridine ether PPAR agonists were synthesized through an ADDP and PS-PPh_3_ modified Mitsunobu protocol, which eliminated significant by-product formation. This method proved to be versatile, efficient and amenable to parallel synthesis.

## Findings

Peroxisome proliferator-activated receptors (PPARs) are pharmaceutical targets of great importance. Their wide-ranging effects on key transcriptional pathways for lipid handling, insulin sensitivity, inflammation and other functions have led to marketed drugs and vast clinical and preclinical research efforts.[1–11]

**Figure 1:**
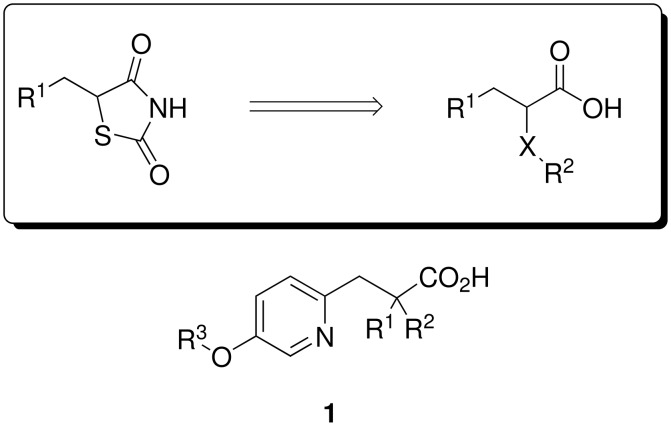
Thiazolidine-2,4-dione mimic & chosen lead scaffold.

In 1991, a series of PPAR analogues were disclosed, which for the first time did not contain a thiazolidine-2,4-dione pharmacophore.[12] These were propanoic acid derivatives with α-substitution to collectively serve as a mimic for the thiazolidine-2,4-dione ring. Based on the above and a knowledge of PPAR ligands publicly disclosed, we wished to synthesize compounds represented by the general structure **1** (Figure 1). Aromatic ethers are structural motifs found in many naturally occurring molecules and compounds of medicinal interest.[13] We envisaged the pyridyl ether moiety of **1** to be efficiently formed via Mitsunobu coupling of the requisite pyridinol and alkyl alcohols.[14–17]

**Scheme 1:**
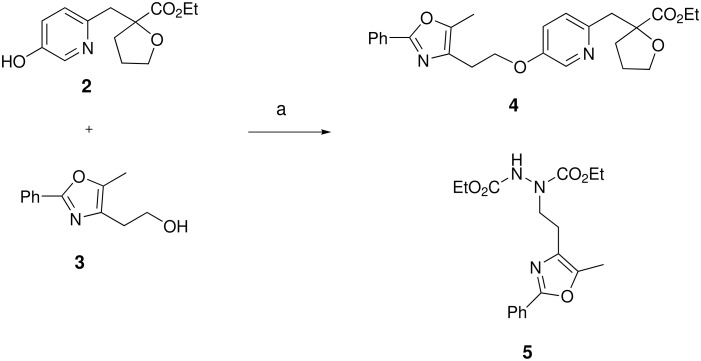
Reagents and conditions: (a) PS-PPh_3_, DEAD, THF, rt, 16 h, 54%.

Our first attempt at the Mitsunobu reaction between pyridinol **2** and alcohol **3**, utilizing a modification of the conditions originally reported by Mitsunobu,[18] afforded pyridyl ether **4** in 54% yield (Scheme 1). Interestingly, the reaction did not reach completion and pyridinol **2** was recovered, despite the fact that it was the limiting reagent. Upon closer examination, compound **5** was observed as a major by-product (46% based on **3**).

**Figure 2:**
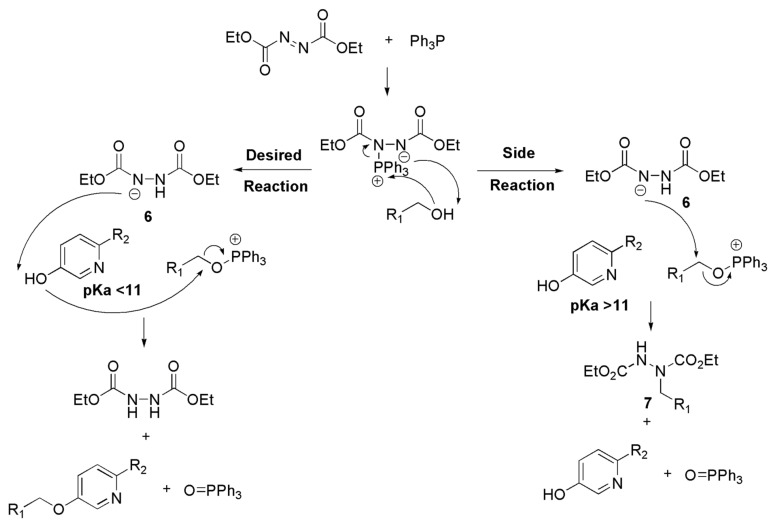
Proposed mechanism of the Mitsunobu reaction.

By-products analogous to **5** have been observed in the literature when diethyl azodicarboxylate (DEAD) is used in certain Mitsunobu reactions.[18–19] This by-product formation is believed to be dependent on the pK_a_ of the acidic component (*e.g.* **2**).[18] If the phenol has a pK_a_ > 11, the yield is considerably lower; and with the phenol having pK_a_ > 13, the desired reaction does not occur. The hydrazo anion **6**, in these cases, attacks the alkoxyphosphonium directly to afford alkylated hydrazine derivative **7** as the by-product (side reaction in Figure 2),[19] since anion **6** is not efficient in deprotonating the weakly acidic phenol.

One way to improve the above redox system is to enhance the basicity of anion **6** by the replacement of the alkoxy group OEt in DEAD with strong electron donating groups such as NR_2_.[16] Thus, 1,1'-(azodicarbonyl)dipiperidine (ADDP),[20] 4,7-dimethyl-3,5,7-hexahydro-1,2,4,7-tetrazocin-3,8-dione (DHTD),[21] and N,N,N',N'-tetramethylazodicarboxamide (TMAD)[22] have been developed as new reagents in combination with tributyl phosphine (TBP).

**Scheme 2:**
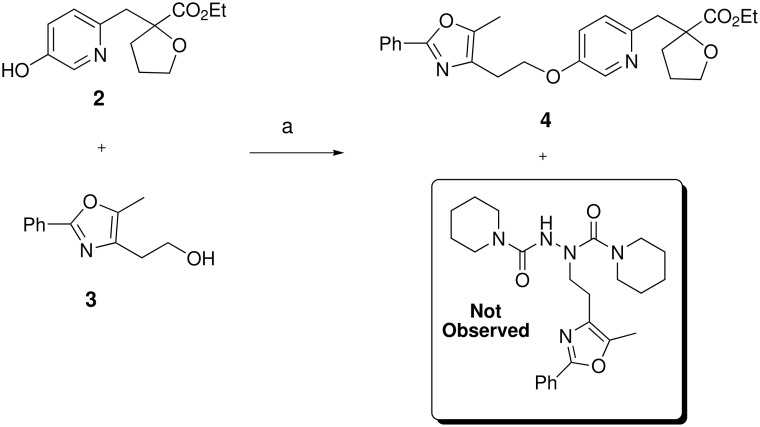
Reagents and conditions: (a) PS-PPh_3_, ADDP, THF, rt, 16 h, 81%.

We initially chose ADDP, due to its commercial availability and low cost. The original reference utilized ADDP and TBP in benzene,[20] but due to safety and ease of handling we chose to keep PS-PPh_3_ and THF. Our first attempt was successful and no by-product **8** was observed in the reaction (Scheme 2) [see Supporting Information File 1 and Supporting Information File 2]. With the above result in hand, we then pursued a variety of targets by performing the modified Mitsunobu reaction of **2** and a variety of primary alcohols (Table 1). In general, a variety of diverse alcohols afforded the expected products in excellent yield. As expected, oxazoles, thiazoles, pyrazoles, and pyridines are tolerated in this chemistry. In a limited number of cases, functionality (*e.g.* basic amines, benzimidazoles, indoles, etc.) caused no reaction to occur and only recovered starting materials were isolated (data not shown).

**Table 1:** Modified Mitsunobu coupling of pyridinol **2** and a variety of primary alcohols^a^

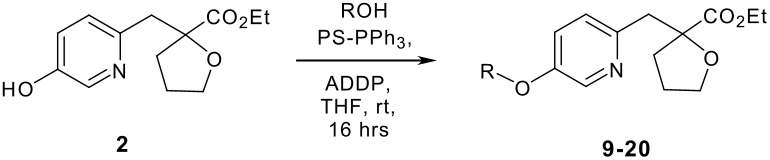							
***Entry***	***Alcohol***	***Product***	***Yield (%)***	***Entry***	***Alcohol***	***Product***	***Yield (%)***

1	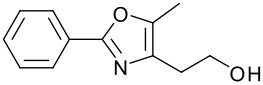	**9**	81	7	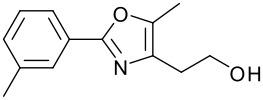	**15**	79
2	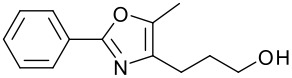	**10**	78	8	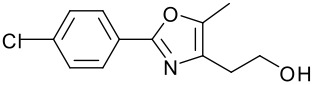	**16**	85
3	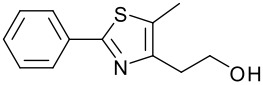	**11**	80	9	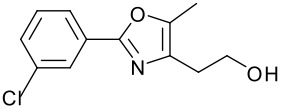	**17**	77
4	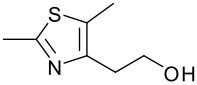	**12**	83	10	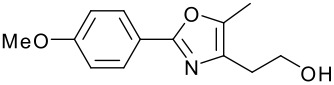	**18**	80
5	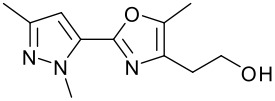	**13**	71	11	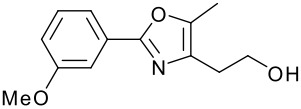	**19**	72
6	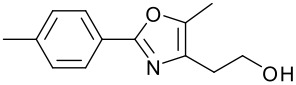	**14**	81	12	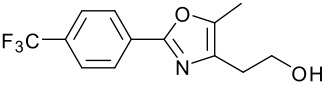	**20**	76

^a^ Reactions were run using 0.5 mmol pyridinol, 0.55 mmol alcohol, 0.75 mmol PS-PPh_3_, 0.75 mmol ADDP and 5.5 mL tetrahydrofuran.

We then shifted our attention to variation of the pyridinol, whilst holding constant the 2-(5-methyl-2-phenyl-1,3-oxazol-4-yl)ethanol reactant **3** (Table 2). As expected, variation of the 2-substituent of the pyridine ring resulted in equally high yields.

**Table 2:** Modified Mitsunobu coupling of alcohol **3** and a variety of pyridinols^a^

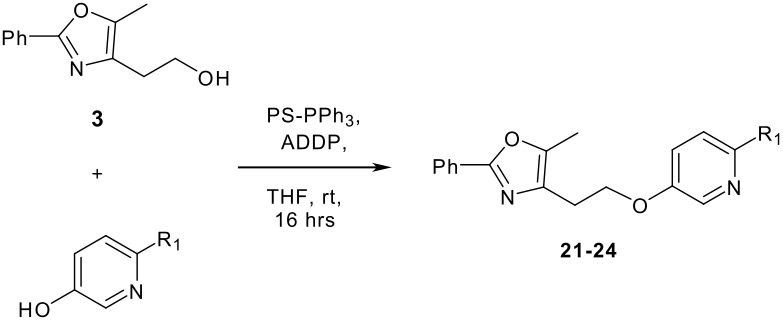							
***Entry***	***Pyridinol***	***Product***	***Yield (%)***	***Entry***	***Pyridinol***	***Product***	***Yield (%)***

1	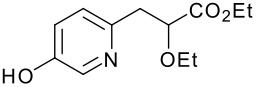	**21**	86	3	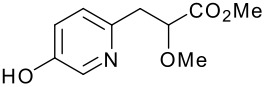	**23**	84
2	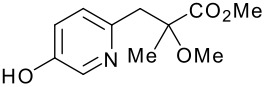	**22**	95	4	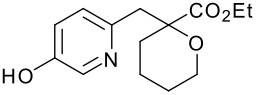	**24**	78

^a^ Reactions were run using 0.5 mmol pyridinol, 0.55 mmol alcohol, 0.75 mmol PS-PPh_3_, 0.75 mmol ADDP and 5.5 mL tetrahydrofuran.

**Scheme 3:**
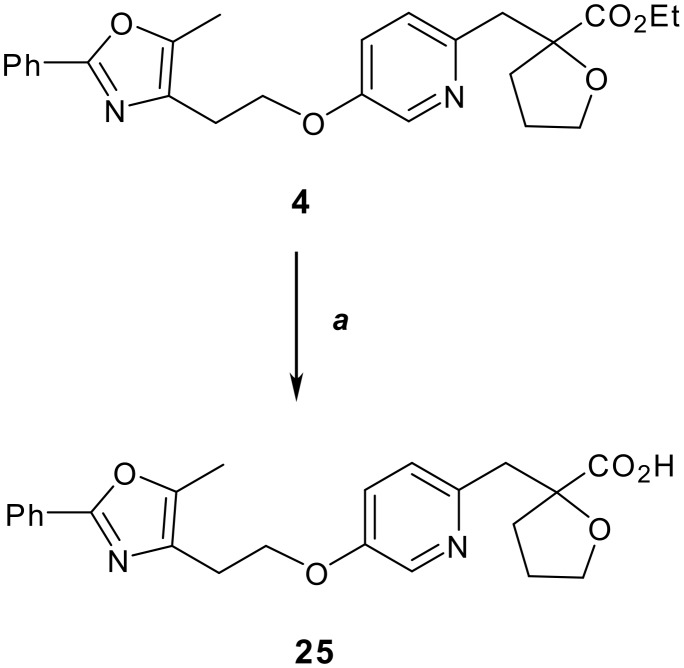
Reagents and conditions: (a) 1 N aq. NaOH, MeCN, 100°C μW, 10 mins, 96%.

Having efficiently synthesized a diverse set of intermediate esters, we then sought an expedient method for obtaining the final carboxylic acids. We opted for a microwave-assisted procedure for this basic hydrolysis step. As shown in Scheme 3, the carboxylic acids (*e.g.* **25**) could be obtained in a matter of minutes [see Supporting Information File 1 and Supporting Information File 2]. The significant reduction in reaction time resulted in a productivity enhancement due to increased sample processing. A number of the other esters described in this manuscript were also subjected to these conditions and all afforded the pure carboxylic acids in 82–100% yield.

In summary, we have developed a modified Mitsunobu protocol utilizing 1,1'-(azodicarbonyl)dipiperidine (ADDP) and polymer-supported triphenylphosphine (PS-PPh_3_). Employment of this method resulted in a rapid, convenient, and high-yielding two step protocol for the preparation of PPAR agonists. In particular, the modified Mitsunobu coupling of pyridinols and alcohols proved to be versatile, efficient and amenable to parallel synthesis. A full account of the medicinal chemistry of these compounds will be given elsewhere.

## Supporting Information

File 1 Supporting Information. Experimental procedures and data for all novel compounds described in this manuscript.

File 2 Auxiliary Data. Auxiliary data for all novel compounds described in this manuscript.
