# Supplementary material for: ADDP and PS-PPh3: an efficient Mitsunobu protocol for the preparation of pyridine ether PPAR agonists
Source: Beilstein J Org Chem. 2006 Oct 31;2:21. doi: 10.1186/1860-5397-2-21 (PMC1705810; doi:10.1186/1860-5397-2-21)
Supplement: File 1 — Supporting Information. Experimental procedures and data for all novel compounds described in this manuscript. [file Beilstein_J_Org_Chem-02-21-s001.doc]

# ADDP and PS-PPh_3_: An efficient Mitsunobu protocol for the preparation of pyridine ether PPAR agonists

Paul S. Humphries*, Quyen-Quyen T. Do, David M. Wilhite

Pfizer Global R&D, Department of Medicinal Chemistry, 10614 Science Center Drive, San Diego, CA 92121, USA

**Supporting Information**

Proton magnetic resonance (^1^H NMR) spectra were determined using a Bruker Avance 300 DPX or Bruker Avance 400 DRX spectrometer operating at a field strength of 300 or 400 megahertz (MHz). Chemical shifts are reported in parts per million (ppm) downfield from an internal tetramethylsilane standard. Alternatively, ^1^H NMR spectra were referenced to residual protic solvent signals as follows: CHCl_3_ = 7.26 ppm; DMSO = 2.49 ppm; CH_3_OH = 3.31 ppm. Peak multiplicities are designated as follows: s = singlet; d = doublet; dd = doublet of doublets; t = triplet; q = quartet; br = broad resonance; and m = multiplet. Coupling constants are given in Hertz. Elemental microanalyses were performed by Atlantic Microlab Inc. (Norcross, GA) and gave results for the elements stated within ±0.4% of the theoretical values. Flash column chromatography was performed using Biotage disposable silica columns. Analytical thin layer chromatography (TLC) was performed using precoated sheets of Silica 60 F_254_ (Merck Art 5719). Semi-preparative HPLC samples were run on a Gilson LC3D system fitted with a 21.2 mm x 250 mm C8 column. Ramps were optimized for each compound with a CH_3_CN/H_2_O solvent system. All reactions were performed in septum-sealed flasks under a slight positive pressure of nitrogen, unless otherwise noted. All commercial reagents were used as received from their respective suppliers. Mass spectra were measured using either electrospray (EI) or fast atom bombardment (FAB) ionization techniques.

**General procedure for the modified Mitsunobu synthesis of pyridyl ethers.**

To a solution of pyridinol (0.92 mmol), alcohol (1.01 mmol) and polymer-supported triphenylphosphine (Fluka; 3mmol/g loading) (1.38 mmol) in anhydrous tetrahydrofuran (10 mL), under an atmosphere of nitrogen, was added a solution of 1,1’-(azodicarbonyl)dipiperidine (1.38 mmol) in anhydrous tetrahydrofuran (1 mL) dropwise. The resulting solution was stirred at ambient temperature for 16 hours and the volatiles removed *in vacuo*. The residue was then purified (Biotage Sp4 – Hexanes to Ethyl Acetate over 10 column volumes) by silica gel chromatography to afford the required product.

**Ethyl 2-({5-[2-(5-methyl-2-phenyl-1,3-oxazol-4-yl)ethoxy]pyridin-2-yl}methyl)tetrahydro-furan-2-carboxylate (9)**

LRMS (*m/z*): 437 (M+H)^+^; ^1^H NMR (300 MHz, CDCl_3_) δ 8.20 (d, *J*=2.8 Hz, 1H), 7.99 (d, *J*=2.5 Hz, 1H), 7.96 (d, *J*=1.7 Hz, 1H), 7.70-7.64 (m, 1H), 7.49-7.39 (m, 2H), 7.19 (d, *J*=8.5 Hz, 1H), 7.11 (dd, *J*=3.0, 8.5 Hz, 1H), 4.26 (t, *J*=6.6 Hz, 2H), 4.17 (q, *J*=7.2 Hz, 2H), 3.95-3.81 (m, 2H), 3.33 (d, *J*=13.8 Hz, 1H), 3.16 (d, *J*=13.8 Hz, 1H), 2.98 (t, *J*=6.6 Hz, 2H), 2.37 (s, 3H), 2.34-2.22 (m, 1H), 2.09-2.00 (m, 1H), 1.87-1.76 (m, 1H), 1.72-1.62 (m, 1H), 1.23 (t, *J*=7.2 Hz, 3H).

**Ethyl 2-({5-[3-(5-methyl-2-phenyl-1,3-oxazol-4-yl)propoxy]pyridin-2-yl}methyl)tetrahydro-furan-2-carboxylate (10)**

LRMS (*m/z*): 451 (M+H)^+^; ^1^H NMR (300 MHz, CDCl_3_) δ 8.19 (d, *J*=2.8 Hz, 1H), 7.97-7.93 (m, 2H), 7.42-7.39 (m, 3H), 7.18 (d, *J*=8.5 Hz, 1H), 7.10 (dd, *J*=2.8, 8.5 Hz, 1H), 4.16 (q, *J*=7.2 Hz, 2H), 3.98 (t, *J*=6.0 Hz, 2H), 3.92-3.82 (m, 2H), 3.33 (d, *J*=13.8 Hz, 1H), 3.14 (d, *J*=13.9 Hz, 1H), 2.67 (t, *J*=6.0 Hz, 2H), 2.26 (s, 3H), 2.19-2.10 (m, 2H), 1.91-1.63 (m, 4H), 1.22 (t, *J*=7.2 Hz, 3H).

**Ethyl 2-({5-[2-(5-methyl-2-phenyl-1,3-thiazol-4-yl)ethoxy]pyridin-2-yl}methyl)tetrahydro-furan-2-carboxylate (11)**

LRMS (*m/z*): 453 (M+H)^+^; ^1^H NMR (400 MHz, CDCl_3_) δ 8.20 (d, *J*=2.6 Hz, 1H), 7.78-7.75 (m, 2H), 7.37-7.32 (m, 4H), 7.28 (d, *J*=8.6 Hz, 1H), 4.24 (t, *J*=6.6 Hz, 2H), 4.18 (q, *J*=7.1 Hz, 2H), 3.82-3.71 (m, 2H), 3.32 (d, *J* =13.6 Hz, 1H), 3.15 (d, *J*=13.6 Hz, 1H), 2.97 (t, *J*=6.6 Hz, 2H), 2.36 (s, 3H), 2.36-2.34 (m, 1H), 2.18-2.11 (m, 1H), 1.94-1.87 (m, 1H), 1.78-1.68 (m, 1H), 1.22 (t, *J*=7.2 Hz, 3H).

**Ethyl 2-({5-[2-(2,5-dimethyl-1,3-thiazol-4-yl)ethoxy]pyridin-2-yl}methyl)tetrahydrofuran-2-carboxylate (12)**

LRMS (*m/z*): 391 (M+H)^+^; ^1^H NMR (400 MHz, CDCl_3_) δ 8.19 (dd, *J*=1.5, 2.0 Hz, 1H), 7.08-7.07 (m, 2H), 4.25 (t, *J*=6.6 Hz, 2H), 4.16 (q, *J*=7.0 Hz, 2H), 3.96-3.82 (m, 2H), 3.34 (d, *J*=3.0, 8.5 Hz, 1H), 3.17 (d, *J*=13.8 Hz, 1H), 2.99 (t, *J*=6.6 Hz, 2H), 2.58 (s, 3H), 2.38 (s, 3H), 2.35-2.23 (m, 1H), 2.10-2.01 (m, 1H), 1.88-1.77 (m, 1H), 1.73-1.63 (m, 1H), 1.21 (t, *J*=7.1 Hz, 3H).

**Ethyl 2-[(5-{2-[2-(1,3-dimethyl-1*H*-pyrazol-5-yl)-5-methyl-1,3-oxazol-4-yl]ethoxy}pyridin-2-yl)methyl]tetrahydrofuran-2-carboxylate (13)**

LRMS (*m/z*): 455 (M+H)^+^; ^1^H NMR (400 MHz, CDCl_3_) δ 8.22 (d, *J*=2.6 Hz, 1H), 7.21 (d, *J*=8.4 Hz, 1H), 7.13 (dd, *J*=2.8, 8.4 Hz, 1H), 6.48 (s, 1H), 4.28 (t, *J*=6.5 Hz, 2H), 4.19 (q, *J*=7.1 Hz, 2H), 4.15 (s, 3H), 3.97-3.83 (m, 2H), 3.33 (d, *J*=13.6 Hz, 1H), 3.18 (d, *J*=13.6 Hz, 1H), 3.00 (t, *J*=6.5 Hz, 2H), 2.39 (s, 3H), 2.36-2.24 (m, 1H), 2.27 (s, 3H), 2.11-2.02 (m, 1H), 1.89-1.78 (m, 1H), 1.74-1.64 (m, 1H), 1.25 (t, *J*=7.1 Hz, 3H).

**Ethyl 2-[(5-{2-[5-methyl-2-(4-methylphenyl)-1,3-oxazol-4-yl]ethoxy}pyridin-2-yl)methyl]tetra-hydrofuran-2-carboxylate (14)**

LRMS (*m/z*): 451 (M+H)^+^; ^1^H NMR (400 MHz, CDCl_3_) δ 8.07 (d, *J*=2.8 Hz, 1H), 7.79 (d, *J*=8.1 Hz, 2H), 7.38 (d, *J*=8.1 Hz, 2H), 7.34 (dd, *J*=3.0, 8.6 Hz, 1H), 7.28 (d, *J*=8.8 Hz, 1H), 4.40 (t, *J*=6.2 Hz, 2H), 4.16 (q, *J*=7.2 Hz, 2H), 3.91-3.85 (m, 2H), 3.49 (d, *J*=14.3 Hz, 1H), 3.14 (d, *J*=14.3 Hz, 1H), 3.04 (t, *J*=6.2 Hz, 2H), 2.35 (s, 3H), 2.31 (s, 3H), 2.30-2.22 (m, 1H), 1.99-1.91 (m, 1H), 1.88-1.72 (m, 2H), 1.22 (t, *J*=7.2 Hz, 3H).

**Ethyl 2-[(5-{2-[5-methyl-2-(3-methylphenyl)-1,3-oxazol-4-yl]ethoxy}pyridin-2-yl)methyl]tetra-hydrofuran-2-carboxylate (15)**

LRMS (*m/z*): 451 (M+H)^+^; ^1^H NMR (400 MHz, CD_3_OD) δ 8.37 (d, *J*=2.6 Hz, 1H), 8.07 (dd, *J*=2.6, 8.9 Hz, 1H), 7.93-7.90 (m, 2H), 7.79 (d, *J*=8.9 Hz, 1H), 7.47-7.40 (m, 2H), 4.41 (t, *J*=6.2 Hz, 2H), 4.17 (q, *J*=7.2 Hz, 2H), 3.92-3.86 (m, 2H), 3.50 (d, *J*=14.3 Hz, 1H), 3.15 (d, *J*=14.3 Hz, 1H), 3.05 (t, *J*=6.2 Hz, 2H), 2.36 (s, 3H), 2.34 (s, 3H), 2.31-2.23 (m, 1H), 2.00-1.92 (m, 1H), 1.89-1.73 (m, 2H), 1.23 (t, *J*=7.2 Hz, 3H).

**Ethyl 2-[(5-{2-[2-(4-chlorophenyl)-5-methyl-1,3-oxazol-4-yl]ethoxy}pyridin-2-yl)methyl]tetra-hydrofuran-2-carboxylate (16)**

LRMS (*m/z*): 471 (M+H)^+^; ^1^H NMR (400 MHz, CDCl_3_) δ 8.04 (d, *J*=2.8 Hz, 1H), 7.85-7.83 (m, 2H), 7.41-7.39 (m, 2H), 7.33 (dd, *J*=3.0, 8.6 Hz, 1H), 7.27 (d, *J*=8.8 Hz, 1H), 4.23 (t, *J*=6.3 Hz, 2H), 4.14 (q, *J*=7.2 Hz, 2H), 3.82-3.70 (m, 2H), 3.17-3.15 (m, 1H), 3.03 (d, *J*=14.2 Hz, 1H), 2.91 (t, *J*=6.3 Hz, 2H), 2.29 (s, 3H), 2.19-2.12 (m, 1H), 1.96-1.88 (m, 1H), 1.78-1.71 (m, 1H), 1.61-1.54 (m, 1H), 1.22 (t, *J*=7.2 Hz, 3H).

**Ethyl 2-[(5-{2-[2-(3-chlorophenyl)-5-methyl-1,3-oxazol-4-yl]ethoxy}pyridin-2-yl)methyl]tetra-hydrofuran-2-carboxylate (17)**

LRMS (*m/z*): 471 (M+H)^+^; ^1^H NMR (400 MHz, CDCl_3_) δ 8.04 (d, *J*=2.8 Hz, 1H), 7.96 (s, 1H), 7.86-7.83 (m, 1H), 7.39-7.36 (m, 2H), 7.33 (dd, *J*=3.0, 8.6 Hz, 1H), 7.27 (d, *J*=8.8 Hz, 1H), 4.23 (t, *J*=6.3 Hz, 2H), 4.14 (q, *J*=7.2 Hz, 2H), 3.82-3.70 (m, 2H), 3.17-3.15 (m, 1H), 3.03 (d, *J*=14.2 Hz, 1H), 2.91 (t, *J*=6.3 Hz, 2H), 2.29 (s, 3H), 2.19-2.12 (m, 1H), 1.96-1.88 (m, 1H), 1.78-1.71 (m, 1H), 1.61-1.54 (m, 1H), 1.22 (t, *J*=7.2 Hz, 3H).

**Ethyl 2-[(5-{2-[2-(4-methoxyphenyl)-5-methyl-1,3-oxazol-4-yl]ethoxy}pyridin-2-yl)methyl]-tetrahydrofuran-2-carboxylate (18)**

LRMS (*m/z*): 467 (M+H)^+^; ^1^H NMR (400 MHz, CD_3_OD) δ 8.01 (d, *J*=1.5 Hz, 1H), 7.80-7.77 (m, 2H), 7.25-7.24 (m, 2H,), 6.93-6.91 (m, 2H), 4.20 (t, *J*=6.3 Hz, 2H), 4.17 (q, *J*=7.2 Hz, 2H), 3.80-3.69 (m, 2H), 3.75 (s, 3H), 3.17-3.15 (m, 1H), 3.01 (d, *J*=13.9 Hz, 1H), 2.88 (t, *J*=6.3 Hz, 2H), 2.26 (s, 3H), 2.17-2.11 (m, 1H), 1.94-1.87 (m, 1H), 1.75-1.66 (m, 1H), 1.58-1.49 (m, 1H), 1.24 (t, *J*=7.2 Hz, 3H).

**Ethyl 2-[(5-{2-[2-(3-methoxyphenyl)-5-methyl-1,3-oxazol-4-yl]ethoxy}pyridin-2-yl)methyl]-tetrahydrofuran-2-carboxylate (19)**

LRMS (*m/z*): 467 (M+H)^+^; ^1^H NMR (400 MHz, CD_3_OD) δ 8.01 (d, *J*=2.3 Hz, 1H), 7.44-7.40 (m, 2H), 7.30-7.22 (m, 3H), 6.94-6.91 (m, 1H), 4.21 (t, *J*=6.4 Hz, 2H), 4.16 (q, *J*=7.2 Hz, 2H), 3.80-3.67 (m, 2H), 3.75 (s, 3H), 3.21-3.17 (m, 1H), 3.01 (d, *J*=13.9 Hz, 1H), 2.90 (t, *J*=6.4 Hz, 2H), 2.28 (s, 3H), 2.17-2.11 (m, 1H), 1.94-1.87 (m, 1H), 1.77-1.67 (m, 1H), 1.60-1.50 (m, 1H), 1.22 (t, *J*=7.2 Hz, 3H).

**Ethyl 2-{[5-(2-{5-methyl-2-[4-(trifluoromethyl)phenyl]-1,3-oxazol-4-yl}ethoxy)pyridin-2-yl]methyl}tetrahydrofuran-2-carboxylate (20)**

LRMS (*m/z*): 505 (M+H)^+^; ^1^H NMR (400 MHz, CDCl_3_) δ 8.05 (d, *J*=2.8 Hz, 1H), 8.00 (d, *J*=8.1 Hz, 2H), 7.70 (d, *J*=8.3 Hz, 2H), 7.33 (dd, *J*=3.0, 8.6 Hz, 1H), 7.27 (d, *J*=8.8 Hz, 1H), 4.23 (t, *J*=6.3 Hz, 2H), 4.14 (q, *J*=7.2 Hz, 2H), 3.82-3.70 (m, 2H), 3.17-3.15 (m, 1H), 3.03 (d, *J*=14.2 Hz, 1H), 2.91 (t, *J*=6.3 Hz, 2H), 2.29 (s, 3H), 2.19-2.12 (m, 1H), 1.96-1.88 (m, 1H), 1.78-1.71 (m, 1H), 1.61-1.54 (m, 1H), 1.22 (t, *J*=7.2 Hz, 3H).

**Ethyl 2-ethoxy-3-{5-[2-(5-methyl-2-phenyl-1,3-oxazol-4-yl)ethoxy]pyridin-2-yl}propanoate (21)**

LRMS (*m/z*): 425 (M+H)^+^; ^1^H NMR (400 MHz, CDCl_3_) δ 8.20 (dd, *J*=1.5, 2.0 Hz, 1H), 7.95 (dd, *J*=1.9, 7.7 Hz, 2H), 7.43-7.36 (m, 3H), 7.10 (d, *J*=1.5 Hz, 2H), 4.25 (t, *J*=6.6 Hz, 2H), 4.23-4.13 (m, 3H), 3.63-3.55 (m, 1H), 3.37-3.27 (m, 1H), 3.15-3.02 (m, 2H), 2.97 (t, *J*=6.7 Hz, 2H), 2.35 (s, 3H), 1.21 (t, *J*=7.2 Hz, 3H), 1.08 (t, *J*=7.1 Hz, 3H).

**Methyl 2-methoxy-2-methyl-3-{5-[2-(5-methyl-2-phenyl-1,3-oxazol-4-yl)ethoxy]pyridin-2-yl}propanoate (22)**

LRMS (*m/z*): 411 (M+H)^+^; ^1^H NMR (400 MHz, CDCl_3_) δ 8.18 (dd, *J*=1.3, 2.3 Hz, 1H), 7.96 (dd, *J*=2.0, 7.6 Hz, 2H), 7.54-7.51 (m, 3H), 7.10-7.08 (m, 2H), 4.25 (t, *J*=6.7 Hz, 2H), 3.29 (s, 3H), 3.15 (s, 2H), 2.96 (t, *J*=6.7 Hz, 2H), 2.36 (s, 3H), 2.32 (s, 3H), 1.38 (s, 3H).

**Methyl 2-methoxy-3-{5-[2-(5-methyl-2-phenyl-1,3-oxazol-4-yl)ethoxy]pyridin-2-yl}propanoate (23)**

LRMS (*m/z*): 397 (M+H)^+^; ^1^H NMR (400 MHz, DMSO-d_6_) δ 8.19 (d, *J*=2.0 Hz, 1H), 7.94 (dd, *J*=1.9, 7.7 Hz, 2H), 7.42-7.35 (m, 3H), 7.09 (d, *J*=1.5 Hz, 2H), 4.44 (t, *J*=6.8 Hz, 2H), 3.88 (dd, *J*=4.8, 7.6 Hz, 1H), 3.22 (s, 3H), 2.92-2.87 (m, 3H), 2.80 (dd, *J*=7.8, 14.2 Hz, 1H), 2.32 (s, 3H), 1.38 (s, 3H).

**Ethyl 2-({5-[2-(5-methyl-2-phenyl-1,3-oxazol-4-yl)ethoxy]pyridin-2-yl}methyl)tetrahydro-2*H*-pyran-2-carboxylate (24)**

LRMS (*m/z*): 451 (M+H)^+^; ^1^H NMR (400 MHz, CDCl_3_) δ 8.17 (d, *J*=2.3 Hz, 1HHGH), 7.96 (dd, *J*=1.9, 7.7 Hz, 2H), 7.43-7.38 (m, 3H), 7.12 (d, *J*=8.1 Hz, 1H), 7.09 (dd, *J*=2.8, 8.6 Hz, 1H), 4.24 (t, *J*=6.7 Hz, 2H), 4.15 (q, *J*=7.2 Hz, 2H), 3.88-3.84 (m, 1H), 3.64 (dt, *J*=3.3, 11.6 Hz, 1H), 3.07 (s, 2H), 2.96 (t, *J*=6.6 Hz, 2H), 2.36 (s, 3H), 2.22-2.18 (m, 1H), 1.52-1.36 (m, 5H), 1.20 (t, *J*=7.1 Hz, 3H).

**General procedure for the microwave-assisted hydrolysis reaction.**

A Smith Process Vial (2-5 mL) was charged, under nitrogen, with a stir bar, ester (0.5 mmol), 1N aq. sodium hydroxide (1.5 mmol) and acetonitrile (2 mL). The reaction vessel was sealed and heated at 100 ^o^C for 10 mins (fixed hold time) in a Biotage Emrys Creator. After cooling, the vessel was uncapped and the reaction mixture acidified to pH 5-7 with 1N aq. hydrochloric acid. The resulting mixture was extracted with ethyl acetate (3 x 15mL) and the combined organic extracts dried (anhydrous magnesium sulfate), filtered and concentrated *in vacuo*. Purification (Biotage Sp4 – Ethyl Acetate to 50% Methanol/Ethyl Acetate over 10 column volumes) by silica gel chromatography afforded the required product.

**2-({5-[2-(5-Methyl-2-phenyl-1,3-oxazol-4-yl)ethoxy]pyridin-2-yl}methyl)tetrahydrofuran-2-carboxylic acid (25)**

LRMS (*m/z*): 408 (M+H)^+^; ^1^H NMR (400 MHz, CD_3_OD) δ 8.37 (d, *J*=2.6 Hz, 1H), 8.07 (dd, *J*=2.6, 8.9 Hz, 1H), 7.93-7.90 (m, 2H), 7.79 (d, *J*=8.9 Hz, 1H), 7.47-7.40 (m, 3H), 4.41 (t, *J*=6.2 Hz, 2H), 3.92-3.86 (m, 2H), 3.50 (d, *J*=14.3 Hz, 1H), 3.15 (d, *J*=14.3 Hz, 1H), 3.05 (t, *J*=6.2 Hz, 2H), 2.36 (s, 3H), 2.31-2.23 (m, 1H), 2.00-1.92 (m, 1H), 1.89-1.73 (m, 2H); Anal. Calcd. For C_23_H_25_N_2_O_5_Cl: C, 62.09; H, 5.66; N, 6.30. Found: C, 61.96; H, 5.75; N, 6.18.
